# Distribution Patterns of Ciliate Diversity in the South China Sea

**DOI:** 10.3389/fmicb.2021.689688

**Published:** 2021-09-03

**Authors:** Weiwei Liu, George B. McManus, Xiaofeng Lin, Honghui Huang, Wenjing Zhang, Yehui Tan

**Affiliations:** ^1^Key Laboratory of Tropical Marine Bio-Resources and Ecology, South China Sea Institute of Oceanology, Chinese Academy of Sciences, Guangzhou, China; ^2^Southern Marine Science and Engineering Guangdong Laboratory (Guangzhou), Guangzhou, China; ^3^Department of Marine Sciences, University of Connecticut, Groton, CT, United States; ^4^Key Laboratory of the Ministry of Education for Coastal and Wetland Ecosystem, College of the Environment and Ecology, Xiamen University, Xiamen, China; ^5^Guangdong Provincial Key Laboratory of Fishery Ecology and Environment, Key Laboratory of South China Sea Fishery Resources Exploitation and Utilization, Ministry of Agriculture and Rural Affairs, P. R. China, South China Sea Fisheries Research Institute, Chinese Academy of Fishery Sciences, Guangzhou, China; ^6^State Key Laboratory of Marine Environmental Science, Marine Biodiversity and Global Change Research Center, Xiamen University, Xiamen, China

**Keywords:** biogeography, community assembly, intertidal, motility, neritic, oceanic, feeding habit

## Abstract

Ciliates are abundant microplankton that are widely distributed in the ocean. In this paper, the distribution patterns of ciliate diversity in the South China Sea (SCS) were analyzed by compiling community data from previous publications. Based on morphological identification, a total of 592 ciliate species have been recorded in the SCS. The ciliate communities in intertidal, neritic and oceanic water areas were compared in terms of taxonomy, motility and feeding habit composition, respectively. Significant community variation was revealed among the three areas, but the difference between the intertidal area and the other two areas was more significant than that between neritic and oceanic areas. The distributions of ciliates within each of the three areas were also analyzed. In the intertidal water, the community was not significantly different among sites but did differ among habitat types. In neritic and oceanic areas, the spatial variation of communities among different sites was clearly observed. Comparison of communities by taxonomic and ecological traits (motility and feeding habit) indicated that these traits similarly revealed the geographical pattern of ciliates on a large scale in the SCS, but to distinguish the community variation on a local scale, taxonomic traits has higher resolution than ecological traits. In addition, we assessed the relative influences of environmental and spatial factors on assembly of ciliate communities in the SCS and found that environmental selection is the major process structuring the taxonomic composition in intertidal water, while spatial processes played significant roles in influencing the taxonomic composition in neritic and oceanic water. Among ecological traits, environmental selection had the most important impact on distributions.

## Introduction

Ciliates are common members of the microplankton, and usually dominate marine microzooplankton communities in terms of both species number and abundance (Azam and Malfatti, 2007; Lynn, 2008; De Vargas et al., 2015). By consuming phytoplankton while also serving as prey for metazoans, these protists act as an intermediate link of energy transfer in planktonic food webs (Pierce and Turner, 1992; Fenchel, 2008).

An increasing number of investigations on diversity and distribution of ciliates have been conducted worldwide (Dolan and Marrasé, 1995; Leakey et al., 1996; Pitta and Giannakourou, 2000; Ota and Taniguchi, 2003; Johansson et al., 2004; Gómez, 2007; Yang et al., 2020). However, one of the most hotly debated issues remains unresolved, i.e., whether or not ciliate distributions are spatially restricted. Some researchers find that global ciliate diversity is relatively low and local diversity covers a very high proportion of global diversity (Fenchel et al., 1997; Fenchel and Finlay, 2004), while others find an extremely high global diversity and that the proportion of the global species pool found locally is only moderate (Pierce and Turner, 1993; Foissner et al., 2008; Agatha, 2011). To address this issue, it is essential to explore the mechanisms that determine the assembly of ciliate communities (Dolan et al., 2007; Doherty et al., 2010). A large number of studies have confirmed that ciliate communities can be influenced by environmental variables including nutrients, salinity, pH, temperature, and biotic interactions (e.g., predators) (Urrutxurtu et al., 2003; Forster et al., 2012; Gimmler et al., 2016; Sun et al., 2017). Meanwhile, spatial factors (dispersal) have also been considered in the study of ciliate community assembly. Any dispersal limitation should lead to a decrease in community similarity with distance (Gong et al., 2015; Zhao et al., 2017; Pan et al., 2020). However, a growing number of studies have indicated that the influence of environmental and spatial variables on ciliates depends on study scale and ecosystem types (Langenheder and Ragnarsson, 2007; Martiny et al., 2011; Soininen et al., 2011; Hanson et al., 2012; Zhang et al., 2018). For example, the ciliate community structure in the mesopelagic zone is mainly controlled by depth and geographic distance (Grattepanche et al., 2016b; Sun et al., 2019), while environmental selection exhibits a greater influence on ciliates than spatial factors in intertidal sandy sediments at continental scale (Pan et al., 2020).

There is a growing interest in the diversity and distributions of ciliates, considered according to their ecological traits (e.g., Green et al., 2008; Soininen et al., 2016). Ecological traits can be more directly linked to species fitness or performance than taxonomical identity can (Laureto et al., 2015). Therefore, to better understand the relationships between communities and the environment, it is necessary to investigate the geographical patterns of ecological trait composition as well as potential mechanisms whereby they may affect community composition (McGill et al., 2006; Villéger et al., 2011).

As the largest semi-enclosed basin in the western Pacific Ocean and the largest marginal sea of China, the South China Sea (SCS) has an expansive area which covers both subtropical and tropical regions (Su, 2004). It hosts strong gradients in physico-chemical environments and is known as an important hotspot of marine biodiversity (Chen et al., 2001; Huang et al., 2004; Li et al., 2017; Sun et al., 2019). Many studies on the diversity and distribution of ciliates have been conducted in the SCS in the last two decades (Su et al., 2007; Liu et al., 2010, 2016a,b; Tan et al., 2010; Wang et al., 2013, 2014; Yu et al., 2014; Wu et al., 2016a,b,c, 2017, 2019; Hu et al., 2019; Huang et al., 2021). However, most of these studies were conducted on a small local scale, such as estuaries, bays, and reef islands; this has resulted in knowledge that is more or less patchy, and the overall ciliate distribution pattern in the SCS is still poorly known.

Here, we collect the available community data on ciliates from the published literature, and analyze distributions in the SCS. Our aims are to quantify the overall spatial patterns by considering the area as a whole, and to check for whether ciliates are regionally restricted or ubiquitously distributed by analyzing the community variations in terms of taxonomy and ecological trait compositions among intertidal, neritic and oceanic areas as well as among different sites within each area. In addition, we also assess the influence of environmental and spatial factors on the geographical distributions of ciliate communities.

## Materials and Methods

### Database

Data were compiled from different sources include one monograph (Hu et al., 2019) and all papers on marine ciliate biodiversity in SCS based on morphological identification from 1991 to 2018 (for the list of papers, see Liu et al., 2021). In total 592 species from 30 investigations were included in the data set (see Supplementary Table S1 in Supplement for a complete list). The community from each investigation was set as one sample in our analyses.

### Community Composition Analyses

Considering spatial environmental variation, we firstly analyzed the large-scale patterns of ciliate community composition based on three geographical area groupings, i.e., the intertidal area (waters covering the area between the low and high tides), the neritic area (beyond the intertidal zone and over the continental shelf), and the oceanic area (from the shelf break to the deep sea) (Liu et al., 2021). Subsequently, in each area, the distribution variations of ciliates on a regional scale were analyzed based on 3–4 subarea groupings (Supplementary Figure S1). Further, for the samples from the intertidal area, five habits types (i.e., estuary, aquaculture pond, beach, mangrove and harbor) were identified and their specific communities were compared. In addition, ciliate abundance were compared among subareas for neritic and oceanic areas, and the abundance data in intertidal areas were not available from the publications and thus were not included in our analyses.

To compare the assemblage composition of each grouping, the samples belonging to same spatial area or subarea were pooled together and the species richness of all taxa were plotted at Class or Subclass levels. Moreover, we clarified the ecological trait composition of ciliates in terms of motility and feeding habits in the areas or subareas. For motility composition, the designation of species as being sessile, vagile or planktonic was made according to the literature (e.g., Foissner, 1992; Foissner et al., 1999; Coppellotti and Matarazzo, 2000; Xu et al., 2009). For feeding habit composition, the species were assigned to five types, which comprised detritivores, bacterivores, algivores, raptors, and non-selectives, according to the original sources in which the species were described, as well as the broader literature (Pratt and Cairns, 1985; Fernandez-Leborans, 2001; Fernandez-Leborans and Fernandez-Fernandez, 2002; Lynn, 2008). Indicator species analysis was performed to identify the species that characterized each areas using the package “Indicspecies” in R (Dufrene and Legendre, 1997). Only species with indicator values (IV) > 0.3 and *p* < 0.05 were considered good indicators.

### Statistical Analyses

In order to facilitate taxonomic consistency between different data sources in community multivariate analyses, species-level data were aggregated to the genus level (presence/absence), which resulted in a data set comprising 207 ciliate genera. Then the genera presence/absence community data were Hellinger transformed prior to the analyses (Peres-Neto et al., 2006). We analyzed the patterns of ciliate communities with non-metric multidimensional scaling (NMDS). Analysis of similarity (ANOSIM) was used to statistically test for significant differences in communities of each area or subarea based on Bray-Curtis dissimilarity with 999 random permutations. The unweighted pair group method with arithmetic mean (UPGMA) hierarchical clustering algorithm was conducted to cluster pairs of communities for each subarea, and we tested robustness of these clusters with jackknife analysis, a non-parametric estimator based on 1,000 randomized subsamples. The relationships between the Bray-Curtis dissimilarity of ciliate communities and geographic distance were analyzed based on Spearman’s rank correlations for each of the three areas.

We explored the impacts of environmental and spatial variables on the communities defined by taxonomic, motility and feeding habit traits. We included 3 environmental variables: Habitat type, Salinity, and Chlorophyll a concentration (Chla). Seven habitat types (i.e., full-open water, semi-open water, estuary, aquaculture pond, beach, mangrove and harbor) were defined as categorical variables. Salinity and Chlorophyll a concentration were averaged for each sample from the original data sources. For some samples with Chlorophyll a data not available, the mean value of last decade was extracted from NASA GSFC.^1^ We followed the approach of the principal coordinates of neighbor matrices (PCNMs) analyses to calculate a set of spatial factors based on the latitude and longitude of each sample. Then, variation partitioning analysis (VPA) was used to evaluate the relative contribution of the environmental and spatial variables in shaping ciliate communities with adjusted R2 coefficients based on redundancy analysis (RDA) or canonical correspondence analysis (CCA). The relative contributions of both components were explained by pure environmental variables, pure spatial variables, and the combined effects of both environment and space. Before the RDA or CCA analysis, a forward selection was conducted to select significant explanatory variables (*P* < 0.05) for further analyses. Mantel tests were also performed to evaluate individual effects of environmental and spatial variables on each ciliate community. All analyses were performed using the “vegan” package in R (R Core Team, 2021). Considering the high similarity of community and environmental characters between neritic and oceanic areas, the data from these two areas were combined to analyze the responses of communities to environmental and spatial variables in open water.

## Results

### Distribution Variation of Ciliates Among Intertidal, Neritic, and Oceanic Water of the SCS

In total, 592 ciliates species, assigned to 14 classes/subclasses, 33 orders, 96 families, and 207 genera, have been detected in the SCS. The subclass Choreotrichia is the most abundant in terms of species number (202 species), which is much higher than the second most abundant subclass, Oligotrichia (84 species) (Supplementary Figure S2).

**FIGURE 1:**
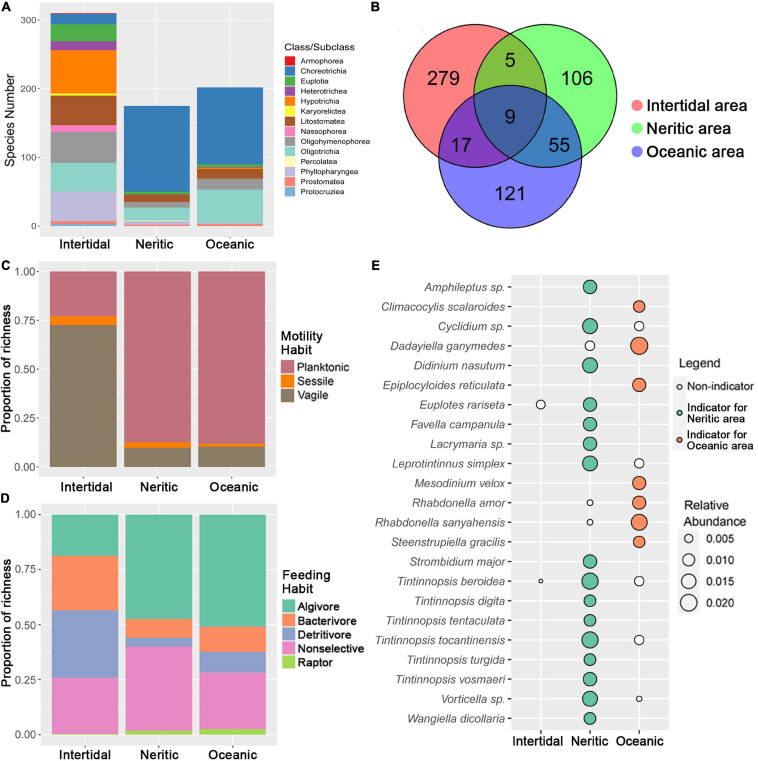
Comparison of ciliate diversity and composition among the three areas in SCS. **(A)** Taxonomy composition patterns of ciliates at class/subclass level in the three areas. **(B)** Venn diagram showing the number of species that are unique and shared among the three areas. **(C)** Proportion of motility habit grouping of ciliates in the three areas. **(D)** Proportion of feeding habit grouping of ciliates in the three areas. **(E)** Indicator species in the three areas (no indicator species was identified in the Intertidal area); the size of the bubble indicates the relative abundance of each species in each area.

The comparison of species richness among the three areas revealed that the intertidal area possesses a remarkably higher number of ciliate species than neritic and oceanic areas. A Venn diagram shows that the number of species found only in one area is dramatically higher than that of shared species (only 9 species were found in all three areas), and the number of species shared by neritic and oceanic communities is greater than those shared between intertidal and other two areas (Figure 1B). The taxonomic compositions of ciliates in neritic and oceanic areas are generally similar to each other at class level due to a high proportion of subclass Choreotrichia, but significantly differ from that of intertidal areas in which the proportions of many classes are similar (Figure 1A). In terms of the motility and feeding habits, intertidal communities also displayed clear differences from other two areas. For motility-based composition, the proportion of planktonic species is much lower than that of vagile species in the intertidal area, while on the other hand in neritic and oceanic areas, the vagiles represented the least abundant motility group (Figure 1C). For feeding habit composition, the abundances of most feeding types are generally equal in intertidal water except raptors, while the proportions of algivores and non-selectives are notably lower than others in neritic and oceanic areas (Figure 1D). In addition, we observed a variation in indicator species in the three areas (Figure 1E), i.e., no indicator species was found in the intertidal area, whereas many *Tintinnopsis* species were identified as the indicators for the neritic area, and rare tintinnids with hyaline loricas constitute the indicators of the oceanic area.

**FIGURE 2:**
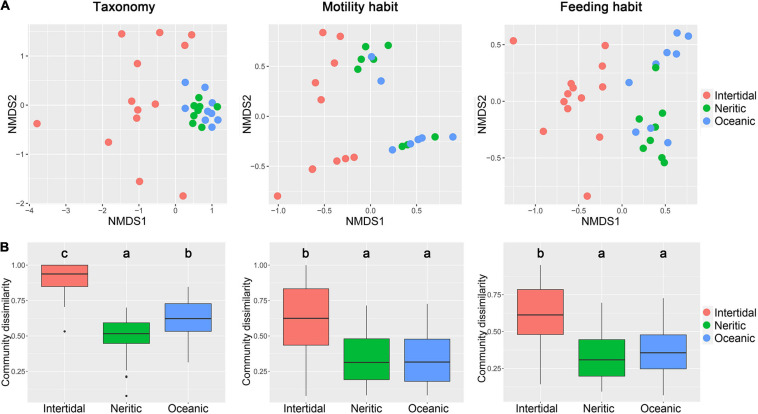
Comparison of ciliate communities in terms of taxonomy, motility, and feeding habits compositions among the three areas in the SCS. **(A)** Non-metric multidimensional scaling (NMDS) ordination of samples in the three areas based on Bray–Curtis dissimilarity. **(B)** Dissimilarity of ciliate communities among the samples in each area.

The similarity analyses revealed that communities exhibited significant differences in the three areas in terms of taxonomy, motility and feeding habits (ANOSIM, *P* < 0.01, Table 1). However, the pairwise tests of the three areas showed that the communities from neritic and oceanic areas cannot be separated by motility and feeding habits, but were significantly different for taxonomic composition. In addition, for all three kinds of community composition, the NMDS indicated that the communities in neritic and oceanic water cluster together, and separated from intertidal communities, of which most samples were widely scattered in the plot (Figure 2A). The dissimilarity of ciliate communities among the samples in intertidal water were significantly higher than neritic and oceanic water in terms of taxonomy, motility and feeding habits compositions, but the differences between neritic and oceanic areas were not significant for motility and feeding habits compositions (Figure 2B).

**TABLE 1:** Analysis of similarities (ANOSIM) of ciliate communities among groupings.

	**Taxonomy**		**Motility**		**Feeding habit**	
	***r***	***P***	***r***	***P***	***r***	***P***
Three areas in SCS	0.215**	0.003	0.348**	<0.001	0.230**	0.002
Intertidal vs. Neritic area	0.216*	0.018	0.448**	0.002	0.260**	0.005
Neritic vs. Oceanic area	0.293**	0.003	−0.077	0.877	0.025	0.322
Intertidal vs. Oceanic area	0.338**	0.002	0.544**	0.002	0.359**	0.002
Subareas in intertidal area	−0.159	0.907	−0.175	0.955	−0.183	0.939
Habitats in intertidal area	0.384**	0.005	0.142	0.213	0.115	0.246
Subareas in neritic area	0.458*	0.014	0.271	0.148	0.292	0.116
Subareas in oceanic area	0.588**	0.008	0.346	0.071	0.246	0.106

*Correlation coefficient (r) and significance levels (P) are indicated.*

*^∗∗^P < 0.01; ^∗^P < 0.05.*

### Distribution Patterns of Ciliate Diversity in Intertidal Water of the SCS

For ciliates in intertidal water, the cluster analyses showed that the taxonomy-based communities that clustered together did not come from the same geographic subarea but from similar habitats (Supplementary Figure S3). This is in accord with the ANOSIM analyses (Table 1), in which the communities in the three intertidal subareas could not be distinguished (*r* = −0.159, *P* = 0.907), but the communities grouped by habitat types were clearly different (*P* = 0.005). Therefore, subsequent community analyses were all conducted based on habitat grouping. However, for motility- and feeding habits-based communities, the ANOSIM analyses did not reveal community variations in either subareas or habitats (*P* > 0.1, Table 1).

**FIGURE 3:**
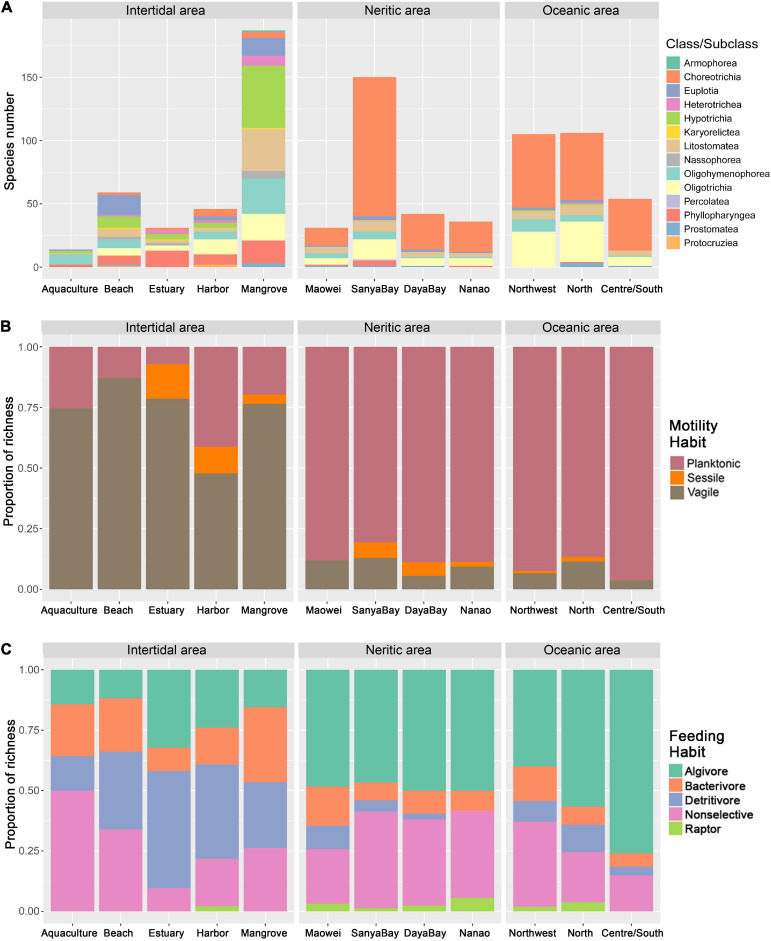
Community variations of ciliate diversity among the subareas in SCS. **(A)** Comparison of ciliate species richness and composition at the class/subclass level among the subgroups. **(B)** Relative proportion of motility habit groupings among the subgroups. **(C)** Relative proportion of feeding habit groupings among the subgroups.

Among the five habitats, the species number detected in mangroves is much higher than in the other habitats, while that in aquaculture ponds is lowest (Figure 3A). For the taxonomy (Figure 3A), motility (Figure 3B), and feeding habits (Figure 3C) based communities, obvious differences can be found among the habitats. Taking feeding habit composition as an example, the algivores and detritivores represent the dominant feeding types in both estuaries and harbors, and the non-selective omnivores dominated the ciliate communities in aquaculture ponds and beach habitats, while the proportion of bacterivores in mangroves is much higher than that in other habits.

**FIGURE 4:**
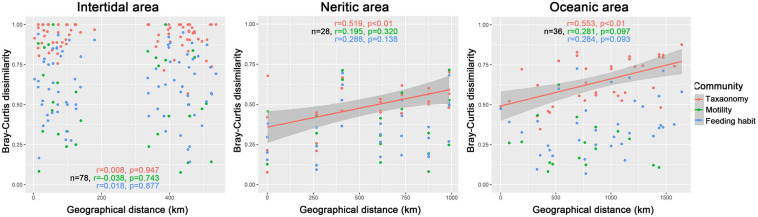
Spearman’s rank correlations between the pairwise Bray–Curtis dissimilarity of communities and the geographical distance of samples in intertidal, neritic, and oceanic water. The n is the number of comparisons for samples, the r is the correlation coefficient for taxonomy (red text and symbols), motility (green text and symbols), and feeding (blue text and symbols) habits compositions.

The Spearman correlograms comparing community dissimilarity and geographical distances among samples did not indicate significant correlations with distance for all taxonomy, motility and feeding habit communities (Figure 4), which means that the community dissimilarity between any two samples did not vary with increasing distance.

### Distribution Patterns of Ciliate Diversity in Neritic Water of the SCS

In neritic areas, the taxonomy-based ciliate communities exhibited striking differences among the four subareas in the ANOSIM analyses (*r* = 0.458, *P* = 0.014, Table 1). In the cluster analyses, the communities from same subarea generally clustered together except for Sanya Bay (Supplementary Figure S3). However, for communities defined by motility and feeding habits, variations in subareas were not distinguished by the ANOSIM analyses (Table 1).

**FIGURE 5:**
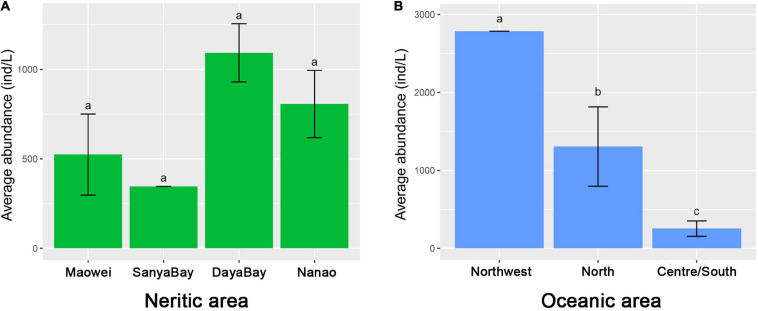
Comparison of ciliate abundances among the subareas in neashore and oceanic areas of SCS. **(A)** Average abundance of ciliates in the four subareas in neritic water. **(B)** Average abundance of ciliates in the four subareas in oceanic water.

Communities defined by feeding habits showed that the relative proportions of most feeding types in the Nanao, Sanya Bay, and Daya Bay are similar, but clearly different from those in Maowei where the proportions of bacterivores and detritivores are higher than in the other three subareas and the proportion of non-selective omnivores is lower (Figure 3C). The motility habit compositions generally are similar among the four subareas (Figure 3B). In addition, the species richness of ciliates in Sanya Bay is remarkably higher than other subareas and that in Maowei is lowest (Figure 3A). In terms of average abundances of ciliates, the highest value occurred in Daya Bay, followed by Nanao and Maowei, and the lowest value was found in Sanya Bay (Figure 5).

For taxonomy-based communities, there were significant and positive relationships between geographical distance and dissimilarity of ciliate communities (Figure 4), while for both motility and feeding habit communities, no significant relationships were exhibited between geographical distance and dissimilarity (Figure 4).

### Distribution Patterns of Ciliate Diversity in Oceanic Water of the SCS

In oceanic water, the ANOSIM analyses revealed the differences in ciliate communities among the three subareas. This is clear in terms of taxonomic composition (*R* = 0.588, *P* = 0.008, Table 1), but not significant in terms of motility and feeding habits. In the cluster analyses, the communities from the Northwest and Centre/South subareas generally form their own clades, but those from North SCS nested within other subareas (Supplementary Figure S3).

The feeding habit composition of ciliates displayed clear differences among the three subareas, i.e., the proportion of algivores gradually increased while that of non-selective omnivores decreased from Northwest to Centre/South (Figure 3C). The motility habit compositions are generally similar among the subareas (Figure 3B). For taxonomic composition, the species richness in Centre/South is distinctly lower than that in other subareas (Figure 3A). The average abundance in Centre/South is lowest among the subareas, and that in Northwest is dramatically higher than the others (Figure 5).

For communities in terms of taxonomic composition, significant positive relationships were revealed between the geographical distance and dissimilarity of ciliate communities, whereas the relationships were not significant in terms of motility and feeding habit composition (Figure 4).

### Impacts of Environmental and Spatial Variables on Ciliate Communities in the SCS

The CCA/RDA ordination showed that Habitat type was the environmental factors that significantly affected all communities and subcommunities, and Chla was related to the taxonomic and feeding habit communities of the entire SCS, while Salinity was not related to either community (Supplementary Figure S4). Regarding spatial variables, no variable was found to be significantly related to intertidal community composition, while 1–3 variables were revealed to have relationships with the other communities (Supplementary Figure S4).

The VPA demonstrated that the community variation in terms of taxonomic composition for the entire SCS was related significantly to both environment and space (*P* < 0.05), and the environmental variables had much higher explanatory power for community variation than spatial variables (20.8% vs. 0%) (Table 2). For open water areas, VPA revealed that both environmental and spatial variables had significant (*P* < 0.05) influence on the communities, although the fraction of the variance explained by spatial variables is slightly higher than environmental variables (7.4% vs. 5.9%). For intertidal communities, no significant spatial variable was selected as an explanatory factor, and environmental variables alone had an explanatory power of 4.5% for community variations. Mantel tests showed consistent results with the above analyses, that the correlations between variations in ciliate communities and environmental or spatial variables in both the entire SCS and just open water were significant (*P* < 0.05), whereas the intertidal communities were primarily governed only by environment (Table 3).

**TABLE 2:** Effects of spatial and environmental variables on the ciliate communities in different areas of SCS analyzed by redundancy analysis (RDA).

**Area**	**Effects**	**Explanation**	***P*-values**
Entire SCS	Pure spatial	0	0.038
	Pure environmental	20.8%	0.001
	Shared	0.6%	
Open water (Neritic + Oceanic water)	Pure spatial	7.4%	0.006
	Pure environmental	5.9%	0.001
	Shared	1.1%	
Intertidal water	Pure spatial	−	
	Pure environmental	4.5%	0.007
	Shared		
Entire SCS for motility community	Pure spatial	0	0.025
	Pure environmental	57.3%	0.001
	Shared	12.3%	
Entire SCS for feeding habit community	Pure spatial	11.3%	0.001
	Pure environmental	40.4%	0.001
	Shared	16.0%	

*The p-values were calculated from an ANOVA permutation test with 999 permutations. (−): Variables not selected by forward selection.*

**TABLE 3:** Mantel tests for the correlation between community and spatial, environmental variables using Pearson’s coefficient.

**Area**	**Effects**	**r**	***P*-values**
Entire SCS	Spatial	0.2875	0.004
	Environmental	0.5462	0.001
Open water (Neritic + Oceanic water)	Spatial	0.2250	0.010
	Environmental	0.2445	0.003
Intertidal water	Spatial	−	−
	Environmental	0.2907	0.005
Entire SCS for motility community	Spatial	0.0750	0.132
	Environmental	0.3830	0.001
Entire SCS for feeding habit community	Spatial	0.2150	0.001
	Environmental	0.4330	0.001

*(−): Variables not selected by forward selection.*

For ciliate communities defined by motility composition, the VPA revealed that the community variations in the entire SCS are mostly determined by the environmental variables which by themselves explained up to 57.3% of the variance. The spatial variables by themselves did not explain a significant amount of the variance, although they contributed a 12.3% proportion of explanation together with environmental variables (Table 2). In Mantel tests, community variations were not significantly related to spatial variables but they were to environmental variables (Table 3). For feeding habit composition communities, the environmental variables by themselves exhibited notably higher contributions to the community variation in the entire SCS than the spatial variables did (40.4% vs. 11.3%) in the VPA, and both environmental and spatial variables were found in Mantel tests to be significantly related to the community variations.

## Discussion

### Comparison of Ciliate Diversity Among Intertidal, Neritic, and Oceanic Areas

Our data aggregation found 592 ciliates species recorded in the SCS based on microscropy studies, which is less than some other seas of the world. For instance, 789 planktonic ciliates have been found in the Baltic Sea (Mironova et al., 2009) and 620 in the Caspian (Alekperov, 2007). Apparently, the diversity of ciliates in the SCS is still not well studied, especially in the wide oceanic areas such as the central and southern SCS where the area accounts for 2/3 of SCS but only four investigations have been conducted (Liu et al., 2021). For the taxonomic composition of ciliates, species of the subclasses Choreotrichia and Oligotrichia represent the dominant group in the SCS (Supplementary Figure S2). This group is also found to dominate in other seas like the Yellow Sea in China, the northwest Atlantic, the south Atlantic, the Baltic Sea and, globally, in the Tara Oceans data (Mironova et al., 2009; Santoferrara and Alder, 2009; Song et al., 2009; Agatha, 2011; Gimmler et al., 2016). The high species richness in this group is not surprising, given that it is a common planktonic taxon, widely distributed in all habitats especially open waters and easily collected in surveys (Dolan et al., 2013).

The ciliate communities in intertidal, neritic and oceanic water are clearly different as identified by ANOSIM, suggesting that the variation of communities occurs across a large spatial scale in the SCS. Our further analyses revealed that the community in intertidal water is more distinctive compared with the other two areas. First, the species richness of ciliates in intertidal water was remarkably higher than in the other two areas (Figure 1A). This was in accordance with some previous studies that found alpha diversity of planktonic ciliates decreased with increasing distance from shore (Doherty et al., 2010; Tamura et al., 2011). The high ciliate diversity in intertidal water may be explained by the diverse habitats and heterogeneous environmental characters there, which thus supplies a wide range of niches for ciliates (Liu et al., 2021). Second, the composition patterns of ciliates in intertidal water differs strikingly from other areas in terms of taxonomy, motility and feeding habits (Figures 1A,C,D). Moreover, the number of species shared by intertidal and neritic or oceanic communities is very low (Figure 1B), indicating a high dissimilarity of taxonomic composition at the species level between intertidal and the other two areas. Similar results have also been found for distributions of tintinnids in the northwest Atlantic where distinct assemblage patterns were revealed between intertidal and oceanic areas, and the OTUs found in nearshore areas were nearly or completely undetectable in oceanic areas (Santoferrara et al., 2016, 2018). Third, our NMDS also showed the communities from neritic and oceanic water cluster together, and were clearly separated from intertidal communities. The notable differences in ciliate communities between intertidal and open water areas is likely due to the influence of environmental factors. It is well known that environmental properties are distinctly different between the intertidal and open water, which can create specific environmental niches, and lead to distinct ciliate community composition (Liu et al., 2021). This is confirmed by our motility and feeding habits composition patterns in the three areas. In intertidal water, the seagrasses and reefs supply extensive attachment surfaces for periphytonic (sessile and vagile) ciliates which thus dominate the community. In both neritic and oceanic areas, the open water is lacking in surfaces for attachment and hence more suitable for a planktonic lifestyle, so the proportion of planktonic species is higher than that of other groups. Meanwhile, the diverse habitats in intertidal areas supply diverse food resources for ciliates, and thus more feeding habit types can exist there compared with the neritic and oceanic areas.

It was notable that in NMDS, the samples from intertidal water are separately distributed, while those from neritic and oceanic water cluster together. Further, the community dissimilarity among samples is significantly higher in the intertidal than neritic and oceanic water in terms of taxonomic, motility, and feeding habit communities (Figure 2). Similar results were also found in previous studies on the New England Shelf, where the ciliate communities in nearshore stations were distinct from nearby ones, whereas communities are more similar among samples within midshelf and oceanic stations (Grattepanche et al., 2016a). All these observations indicate that the ciliate communities from distinct sampling sites are more variable in intertidal water compared to open water. Since intertidal waters are generally impacted by various processes such as human activities and river discharge, the environmental characters were significantly heterogeneous there and affected the distributions of ciliates, which thus leads to the patchy distribution of ciliates (Liu et al., 2021). In contrast, the waters from neritic and oceanic areas are more impacted by ocean currents, and are more homogeneous with similar hydrologic characters, and this results in relatively high similarity of communities.

Our results showed the proportion of the subclass Choreotrichia in total species number is significantly higher in both neritic and oceanic than intertidal water (Figure 1A). Since choreotrichs are mostly composed of tintinnid ciliates, this finding is consistent with some conclusions that species richness of tintinnids increased from coastal to oceanic area (Dolan et al., 2013; Li et al., 2016). Although the composition patterns of the neritic and oceanic communities are highly similar at the class level, the indicator species for these two areas revealed their composition difference at the species level (Figure 1E). Their abundances generally match with their biogeographic preferences, especially for tintinnids. For example, the indicators of neritic areas mostly consist of species of the genera *Tintinnopsis*, *Favella*, and *Leprotintinnus*. According to the tintinnid biogeographic categories established by Dolan et al. (2013), these three genera are classified to neritic assemblages because they are restricted to nearshore waters, and this is consistent with our results. In addition, species of the genera *Rhabdonella*, *Epiplocyloides*, and *Climacocylis* were identified as indicators of oceanic water in our study. According to the same tintinnid biogeographic categories (Dolan et al., 2013), these three genera belong to the warm water group. As the oceanic area of SCS located in the tropical zone with higher water temperature, our result confirms their preference for warm water. Besides, from neritic to oceanic areas the indicator tintinnids have shown a switch from species with agglutinated loricae (with mineral particles like species of *Tintinnopsis* and *Leprotintinnus*) to those with hyaline loricae (like species of *Rhabdonella, Epiplocyloides*, and *Climacocylis*). This can be explained by the finding that the agglutinated species require sufficient small mineral particles to facilitate lorica formation and are thus limited to neritic areas where the concentrations of mineral particles are high, whereas for the hyaline tintinnids the mineral particles are not needed to their lorica structuring and thus they prefer oceanic waters (Gold and Morales, 1976; Rassoulzadegan, 1980; Middlebrook et al., 1987; Lynn et al., 1991; Dolan et al., 2013).

### Distribution Variations of Ciliates Among the Subareas in Intertidal, Neritic, and Oceanic Areas

In intertidal water, spatial variation of ciliate communities was not found among sampling regions in both cluster and ANOSIM analyses, but the communities grouped by habitat types were clearly separated (Supplementary Figure S3 and Table 1). Among the habitat type groupings, the composition patterns also exhibited notable variations in our study especially for feeding habit trait (Figure 3). It is widely accepted that the variation of feeding habit composition can be attributed to the responses of ciliate communities to environmental and spatial changes in marine water (Xu et al., 2018, Xu and Soininen, 2019). In our study, for example, the proportion of bacterivores in mangroves is higher than other habitats, which was apparently correlated with the higher bacteria production due to the sufficient accumulation of organic detritus in mangroves compared with other habitats. In the estuarine zone, the high inflow of nutrients from freshwater facilitates the growth of phytoplankton, resulting in the high proportion of algivores. Meanwhile, the strong mixture and interaction of river discharge and ocean intrusion in the estuarine zone gives rise to a high concentration of dissolved organic matter, which can explain the high proportion of detritivores (Figure 3C). In addition to community composition, we also found differences in species richness in the five habitat types (Figure 3A). For example, the fewest species were detected in aquaculture ponds, which agrees with most previous studies (Stoeck et al., 2018; Forster et al., 2019), and indicates the high impact of the nutrient enrichment due to the accumulation of waste food and fecal matter, as well as the ecotoxic effects of discharged medicines (Wilson et al., 2009; Burridge et al., 2010; Pawlowski et al., 2014). The mangrove wetlands possess the highest species richness among the five habitats (Figure 3A). This result is well supported by the high number of new ciliate species reported in mangrove wetlands in the past decade (Liu et al., 2017, 2019; Bai et al., 2019; Hu et al., 2019; Song et al., 2019). The reason for the high species richness in mangroves is twofold. First, the mangrove has complex environmental composition which can supply a wide range of ecological niches for ciliates. This can be confirmed by our result for the feeding habit composition (Figure 3C), in which the proportions of all feeding types were mostly even in mangroves, suggesting it is suitable for ciliates with various feeding styles. Second, the water in mangroves is rich in nutrients which leads to sufficient food.

In neritic water, the spatial variations of ciliate communities at a local scale can be clearly identified in cluster and ANOSIM analyses in terms of taxonomy. Regarding the feeding habit community composition, we found that Maowei clearly differs from other subareas by having a higher proportion of bacterivores and detritivores (Figure 3C). Maowei is located in a relatively closed bay where the water interaction with the open ocean is weak but the inflow of freshwater from rivers is strong. These processes contribute to the high concentrations of dissolved organic matter and bacteria, and thus lead to a high growth of bacterivores and detritivores (Liu et al., 2016a). The spatial variations of ciliate communities among the four neritic subareas can also be found in the species richness and abundance. It is notable that the species richness is strikingly high in Sanya Bay (Figure 3A), which can probably be attributed to the suitable habitat for ciliates supplied by extensive distribution of coral reef there (Su et al., 2008; Tan et al., 2010). The communities in Daya Bay and Nanao are higher in abundance than in other subareas (Figure 5). Aquaculture is practiced extensively in Daya Bay and Nanao, and the feed generally increases the concentration of nutrients and phytoplankton as the food source for ciliates, which thus leads to high abundance (Wu et al., 2016b, 2017, 2019).

In oceanic areas, the variation of ciliate communities among the three subareas is seen in the results of cluster and ANOSIM analyses in terms of taxonomy. In addition, the community variations also can be observed in the feeding habit composition, i.e., the proportion of algivores increased but that of other feeding types decreased from north to south (Figure 3C). Since the nutrient level is lower in oceanic water than the shelf/slope, the microplankton ecology in the southern SCS tends to be simpler and the proportion of tintinnids in ciliate assemblages becomes higher compared with those in the northern SCS (Figure 3A). Given that most tintinnids feed on phytoplankton, the high proportion of algivores in southern or central SCS is easily explained. In addition to community composition, abundance and species richness are significantly different at the local scale (Figures 3A, 5). Those values in Central/South SCS are smaller than North and Northwest SCS, suggesting the decrease in ciliate diversity from shelf/slope to the oceanic realm. The same trend was revealed in the southwestern Atlantic where the density of ciliates decreased ca. Twofold from the Argentine shelf to oceanic waters (Santoferrara and Alder, 2009, 2012). In shelf and slope areas, the environmental characters were significantly affected by coastal currents and upwelling, which carried nutrients, and thus sustained a high biomass of phytoplankton or bacteria as food for ciliates (Liu et al., 2010; Wang et al., 2014). The oceanic area is characterized by the oligotrophic and high salinity water with low concentration of Chl-a, thus explaining the low ciliate abundance and diversity.

### Different Mechanisms Shaping Ciliate Communites in Intertidal and Open Waters

We found no variation among sampling regions in intertidal areas (Figure 4), but it was clearly present among habitat groupings (Supplementary Figure S3 and Table 1), which suggests that the influence of habitat types or site-specific environmental conditions on communities is stronger than that of spatial variation. This finding was supported by CCA and VPA results, in which only environmental variables showed significant contributions to the community variation in intertidal water, but no spatial variable corresponds to this variation (Supplementary Figure S4 and Table 2). In neritic and oceanic areas, by contrast, the ciliate communities from different geographic subareas displayed clear variation, and significant and positive relationships between the geographical distance and community dissimilarity were revealed (Figure 4, Supplementary Figure S3, and Table 1). Moreover, VPA showed that a larger explained fraction of the community variation was attributed to the spatial than environmental variables in the open water (Table 2).

A few studies have compared the relative influences of selective and neutral processes for assembly of microbial communities in marine ecosystems (Chen et al., 2017; Mo et al., 2018; Zhang et al., 2018). Selective processes explain the community variation by emphasizing the influence of environmental factors, while neutral processes highlight the roles of spatial factors (Hanson et al., 2012; Zhou and Ning, 2017; Zhang et al., 2018). In our study, the distinct distribution patterns of ciliates between intertidal and open (neritic and oceanic) water could be attributed to these two types of mechanisms, respectively. Our VPA results indicate that environmental selection is the major process structuring the ciliate assemblages in intertidal water, while spatial processes (dispersal) played significant roles in influencing the biogeography of ciliates in neritic and oceanic water. These findings concur with Grattepanche et al. (2016a), who found that distance to the shore has a more powerful role in structuring ciliate assemblages in oceanic water than specific features of the environment do, while the variation of some environments such as biotic factors and water circulation drove the distribution of ciliate communities in nearshore stations. Furthermore, the different mechanisms in open and intertidal waters of SCS might be ascribed to their distinct motility habit composition. In our results, the ciliate community in intertidal water was dominated by the periphytonic (sessile and vagile) group (Figure 1C). Since the movements of periphyton were limited to substrates, local environmental factors might be more important than spatial factors in shaping their biogeography. In contrast, in neritic and oceanic areas the planktonic ciliates, which were characterized by high and random dispersal rate, dominated the community, and thus the dispersal limitation contributed the most in affecting their distribution.

### Comparison of Distributions of Ciliate Communities in Terms of Taxonomy, Motility, and Feeding Habits

Since ecological traits consider organisms as dynamic entities that interact with their environment, trait-based approaches make science more predictive and able to forecast ecosystem alterations occurring under rapid environmental changes (Laureto et al., 2015; Xu and Soininen, 2019). Our study provided an opportunity to evaluate potential significance of taxonomy and ecological traits in clarifying the community variation of ciliates. In our NMDS results, similar distribution patterns of the communities in SCS were found in terms of the taxonomic, motility and feeding habit compositions (Figure 2). The ANOSIM analyses showed that the community variations of ciliates among the intertidal, neritic, and oceanic areas can be clearly distinguished for all taxonomic, motility, and feeding habit compositions. Similarly, both species and trait compositions showed spatial variations of benthic ciliates along the coast of China (Xu and Soininen, 2019). These suggest that taxonomic and ecological traits can give the same result in exploring the geographical patterns of ciliates on a large scale. However, the pairwise comparison of the communities of the three areas showed that the variations between neritic and oceanic areas were not revealed by motility and feeding habits but only by taxonomy (Figure 2B and Table 1). This indicates that the environmental differences of neritic and oceanic areas have no influence to filter the ecological traits of ciliates, but produce a strong effect on their species composition assembly, which can be confirmed by the distinct indicator species in the two areas.

At a local scale, the ciliate taxonomic compositions exhibited significant differences among the habitats or subareas for intertidal, neritic and oceanic areas, while the community variations of motility and feeding habits among the subgroupings were not distinguished (Table 1). Moreover, in neritic and oceanic water, the significant and positive relationship between geographical distance and dissimilarity was exhibited for taxonomy-based communities, while no significant correlations were observed for the motility and feeding habit communities (Figure 3). Our results suggest that taxonomic traits have higher resolution than ecological traits to distinguish the community variation at the local scale. At small spatial scales, ecology trait compositions were influenced by mass effects due to the high dispersal ability of ciliates, resulting in spatially homogenized communities (Xu and Soininen, 2019).

Regarding the influence of environment and space on the community variation of ciliates in the entire SCS, VPA and Mantel tests showed similar results for both taxonomic and ecological traits—that environmental variables exhibited a higher contribution to the community variation than spatial variables did (Tables 2, 3). Moreover, the explanatory power of environmental variables alone for motility and feeding habit community variations was notably higher than for taxonomy-based community variations. Considering that our chosen traits (motility and feeding habits) are closely related to environmental conditions, it is not difficult to understand that environmental selection (habitat) must play an important role in structuring the ecological trait assemblages of ciliates. Similar findings were reported in a study on trait composition of diatom communities, with a stronger correlation with environmental variables compared to spatial factors (Soininen et al., 2016). Therefore, ecological traits are a good choice for clarifying the selective mechanisms of community variation. In addition, our RDA showed that feeding habit communities were also significantly influenced by Chla. Considering that Chla is the important indicator of food sources for ciliates, this correlation between feeding communities and Chla indicates the response of ciliates to food availability, as found in some other studies (Sun et al., 2019; Liu et al., 2021; Yang et al., 2020).

### Future Prospects

The data on ciliate diversity from previous studies in our analyses were all based on morphological identification. Due to the differences among the studies in identifiers’ subjective knowledge of ciliate taxonomy and discrepancies in sample treatment (e.g., the samples were fixed with Lugol’s in some studies and formalin in others), there may be some inconsistencies of taxonomy in our data. To reduce this, species-level data were aggregated to genus level (presence/absence data) in our analyses. To eliminate this issue, future studies should be carried out uniformly as far as possible. For example, samples should be treated following standard protocols, and ciliate species identification should be done by the same investigator with professional taxonomy training or by applying molecular methods (Agatha, 2011; Santoferrara and McManus, 2017).

In addition, since environmental data in some previous studies is unavailable, the relationship between ciliate communities and environmental factors (temperature, salinity, etc.) was not analyzed in the present work. Therefore, further investigation should pay more attention to the influence of environmental factors on ciliates to clarify the deterministic processes relating to ciliate distributions in the SCS.

## Data Availability Statement

The datasets presented in this study can be found in online repositories. The names of the repository/repositories and accession number(s) can be found in the article/Supplementary Material.

## Author Contributions

WL and YT conceived the research. WL wrote the manuscript. GM, XL, HH, and WZ critically reviewed the findings and improved the manuscript. All authors contributed to the article and approved the submitted version.

## Conflict of Interest

The authors declare that the research was conducted in the absence of any commercial or financial relationships that could be construed as a potential conflict of interest.

## Publisher’s Note

All claims expressed in this article are solely those of the authors and do not necessarily represent those of their affiliated organizations, or those of the publisher, the editors and the reviewers. Any product that may be evaluated in this article, or claim that may be made by its manufacturer, is not guaranteed or endorsed by the publisher.

## Footnotes

1
http://oceancolor.gsfc.nasa.gov/
