# Supplementary material for: Distribution Patterns of Ciliate Diversity in the South China Sea
Source: Front Microbiol. 2021 Sep 3;12:689688. doi: 10.3389/fmicb.2021.689688 (PMC8446678; doi:10.3389/fmicb.2021.689688)
Supplement: Supplementary file 1 [file Data_Sheet_1.docx]

**
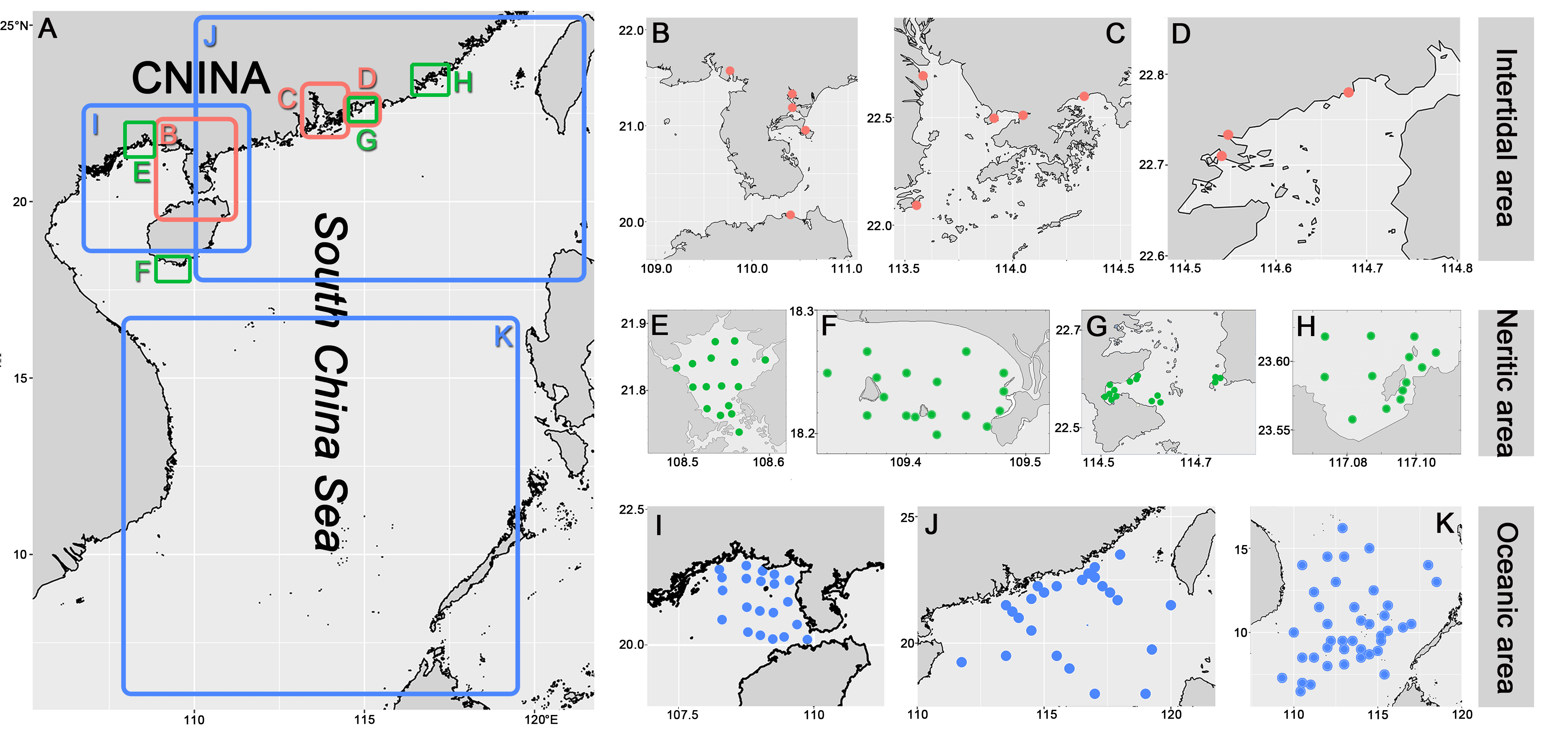
Figure S1.** Locations of all previous studies on the diversity of ciliates in the South China Sea (SCS). (A) The detailed locations of the data resources within each subarea (squares B-K) in SCS. (B) Leizhou Peninsula; (C) Pearl River Outlet; (D) Daya Bay; (E) Maowei; (F) Sanya Bay; (G) Daya Bay; (H) Nanao; (I) Northwestern SCS; (J) Northern SCS; (K) Centre/Southern SCS. The red color indicates the intertidal area, the green color indicates the neritic area, and the blue color indicates the oceanic area. The dots in each subarea show the sampling sites from previous studies (in total 30 investigations in these subareas). The community from each investigation was set as one sample in our analyses.


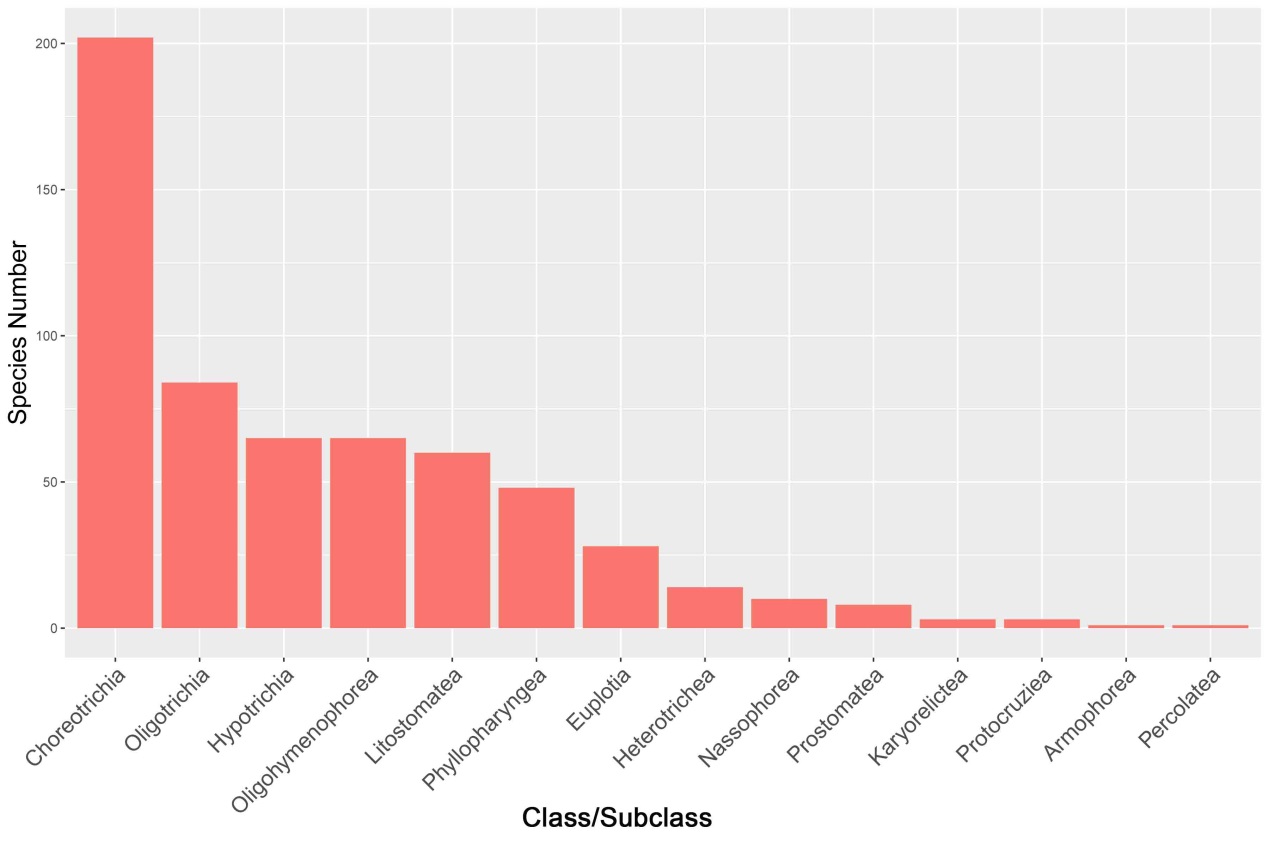


**Figure S2.** Species numbers of ciliates in each identified class found in SCS.

**
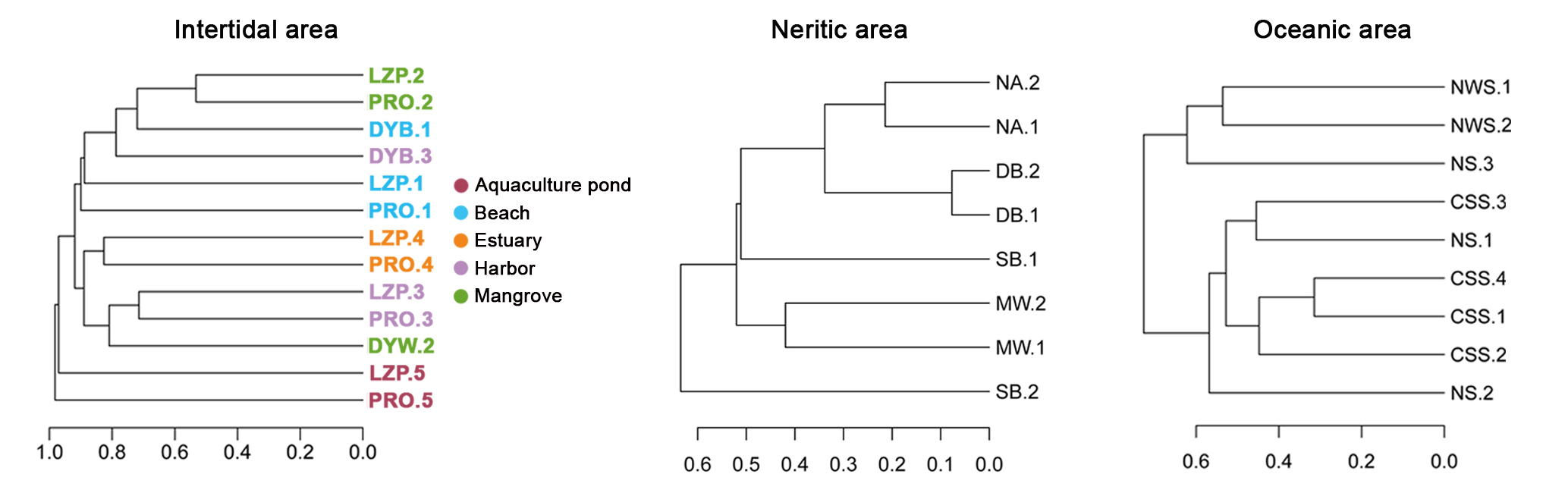
**

**Figure S3.** UPGMA clustering analysis of the samples in Intertidal, Neritic and Oceanic areas based on the Bray-Curtis similarity. Different colors represent different sampling habitats. Abbreviations: LZP. Leizhou Peninsula; PRO. Pearl River Outlet; DYB. Daya Bay; MW. Maowei; SB. Sanya Bay; DB. Daya Bay; NA. Nanao; NWS. Northwestern SCS; NS. Northern SCS; CSS. Centre/Southern SCS.


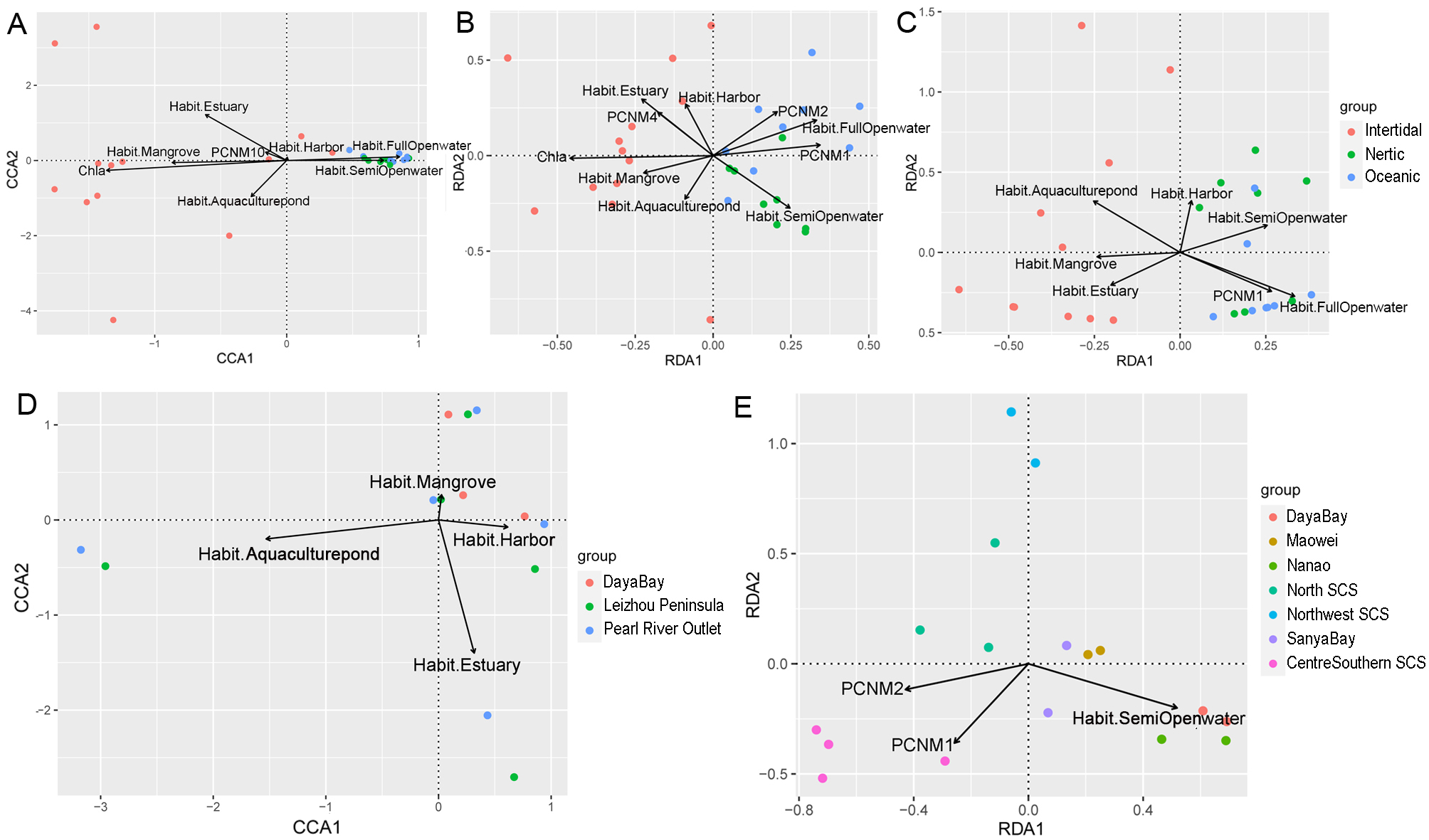


**Figure S4.** CCA and RDA ordination showing the ciliate community in relation to the environmental and spatial variables, respectively. Only statistically significant variables (P<0.01) are shown. (A) For communities in terms of taxonomic composition in entire SCS. (B) For communities in terms of motility habits composition in entire SCS. (C) For communities in terms of feeding habits composition in entire SCS. (D) For communities in terms of taxonomic composition in intertidal area. (E) For communities in terms of taxonomic composition in open water (neritic and oceanic) area.
