# Supplementary material for: Distribution Patterns of Ciliate Diversity in the South China Sea
Source: Front Microbiol. 2021 Sep 3;12:689688. doi: 10.3389/fmicb.2021.689688 (PMC8446678; doi:10.3389/fmicb.2021.689688)
Supplement: Supplementary file 2 [file Data_Sheet_2.docx]

**R Script Used in the Study**

library(vegan)

library(ggplot2)

######ANOSIM for large-scale area#######

data<-read.table("allgenus.txt", header = T, row.names = 1, sep = "\t")

#data<-read.table("Feedcommunity.txt", header = T, row.names = 1, sep = "\t")

#data<-read.table("Biohabitcommunity.txt", header = T, row.names = 1, sep = "\t")

map<-read.table("allmap.txt", header = T, row.names = 1, sep = "\t")

map<-map[colnames(data),]

a<-anosim(t(data),map$Area,permutations = 999,distance = "bray")

summary(a)

plot(a)

########indicspecies##########

library(indicspecies)

allspecies<-read.table("allspecies.txt", header = T, row.names = 1, sep = "\t")

species<-allspecies[,5:34]

indicsp<-as.data.frame(t(species))

sitesmap<-sitesmap[row.names(indicsp),]

groups<-factor(sitesmap$Area,levels = c("onshore","nearshore","offshore"))

indval<-multipatt(indicsp,groups, control = how(nperm = 999))

indvaluesign<-indval$sign

indvalu<-indvaluesign[!is.na(indvaluesign$p.value),]

indvalue<-indvalu[indvalu$p.value<0.05&indvalu$stat>0.3&indvalu$index<4,]

write.table(indvalue,file = "indvalueeachs.txt",sep = "\t",row.names = T,col.names = T,quote = F)

##########ANOSIM and Cluster for intertidal area######

data<-read.table("allgenus.txt", header = T, sep = "\t")

#data<-read.table("Feedcommunity.txt", header = T, row.names = 1, sep = "\t")

#data<-read.table("Biohabitcommunity.txt", header = T, row.names = 1, sep = "\t")

map<-read.table("allmap.txt", header = T, row.names = 1, sep = "\t")

map<-map[map$Area=='onshore',]

onshore<-data[,as.character(row.names(map))]

onshore<-onshore[rowSums(onshore)>0,]

map$Subarea<-factor(map$Subarea)

map$Habit<-factor(map$Habit)

a<-anosim(t(onshore),map$Subarea,permutations = 999,distance = "bray")

summary(a)

plot(a)

b<-anosim(t(onshore),map$Habit,permutations = 999,distance = "bray")

summary(b)

plot(b)

#Cluster

library(ape)

bio<-t(onshore)

distsamp<-vegdist(bio,method = "bray",na.rm = T)

hc3<-hclust(distsamp, method = "average")

tree<-as.dendrogram(hc3)

plot(tree,type="rectangle",horiz=T)

###########ANOSIM and Cluster for neritic and oceanic areas######

nearshore<-data[,as.character(row.names(map[map$Area=="nearshore",]))]

offshore<-data[,as.character(row.names(map[map$Area=="offshore",]))]

mapnear<-map[map$Area=="nearshore",]

mapnear$Subarea<-factor(mapnear$Subarea)

a<-anosim(t(nearshore),mapnear$Subarea,permutations = 999,distance = "bray")

summary(a)

mapoff<-map[map$Area=="offshore",]

mapoff$Subarea<-factor(mapoff$Subarea)

b<-anosim(t(offshore),mapoff$Subarea,permutations = 999,distance = "bray")

summary(b)

#cluster

bio<-t(nearshore)

distsamp<-vegdist(bio,method = "bray",na.rm = T)

hc3<-hclust(distsamp, method = "average")

plot(hc3,hang=-1, cex.lab=0.5)

bio<-t(offshore)

distsamp<-vegdist(bio,method = "bray",na.rm = T)

hc3<-hclust(distsamp, method = "average")

plot(hc3,hang=-1, cex.lab=0.5)

######NMDS for large-scale area#####

data<-read.table("allgenus.txt", header = T, row.names = 1, sep = "\t")

#data<-read.table("Feedcommunity.txt", header = T, row.names = 1, sep = "\t")

#data<-read.table("Biohabitcommunity.txt", header = T, row.names = 1, sep = "\t")

bio<-t(data)

ord<-metaMDS(bio,distance = "bray")

ord$stress

data.scores <- as.data.frame(scores(ord)) #Using the scores function from vegan to extract the site scores and convert to a data.frame

data.scores$site <- rownames(data.scores) # create a column of site names, from the rownames of data.scores

data.scores$grp <- map$Area # add the grp variable created earlier

#data.scores$dp <- map$Location

ggplot() +geom_point(data=data.scores,aes(x=NMDS1,y=NMDS2,colour=grp),size=3) +

# scale_shape_manual(values=c(15:18,7:8,10))+

theme(axis.text = element_text(size=15),legend.title=element_blank(),axis.title=element_text(size=18),strip.text = element_text(size=18),legend.text = element_text(size=15))+

labs(x ="NMDS1",y="NMDS2")

######CCA/RDA+VPA+Manteltest#####

geo2<-read.csv("geo_pcnm.csv",row.names = 1)

env<-read.table("allmap.txt", header = T, row.names = 1, sep = "\t")

geo2<-geo2[row.names(env),]

env<-cbind(geo2,env[, c(5,11:12)])

#1.Taxonomic community for entire SCS

data<-read.table("allgenus.txt", header = T, row.names = 1, sep = "\t")

bio<-data.frame(t(data))

bio<-bio[,colSums(bio)!=0]

bio<-decostand(bio,"hellinger")

decorana(bio) #axis length=8.88

envn<-env[row.names(bio),]

pcnm1<-envn[,1:11] #pcnm10

envnfwda<-ordistep(cca(bio~1,pcnm1),scope = formula(cca(bio~.,pcnm1)),direction = "forward",pstep=1000)

envn1<-envn[,12:14] #Chla+Habit

envnfwda<-ordistep(cca(bio~1,envn1),scope = formula(cca(bio~.,envn1)),direction = "forward",pstep=1000)

environ<-envn[, c(10,12,14)]

ccae<-cca(bio, environ)

ccae

plot(ccae)

location<-data.frame(envn[,c(10)]) #pcnm10

habit<-data.frame(envn[,c(12,15)]) #Chla+Habit

ccal<-cca(bio,location)

anova.cca(ccal) #0.04

ccah<-cca(bio,habit)

anova.cca(ccah) #0.001

winvarpart<-varpart(bio, location, habit)

winvarpart

#Mantel test

location.dis<-vegdist(location,"euclidean")

mantel(vegdist(bio,"bray"),location.dis)

habit.dis<-vegdist(habit,"euclidean")

mantel(vegdist(bio,"bray"),habit.dis)

#2.Taxonomic community for intertidal area

data<-read.table("allgenus.txt", header = T, row.names = 1, sep = "\t")

bio<-data.frame(t(data[,1:13]))

bio<-bio[,colSums(bio)!=0]

bio<-decostand(bio,"hellinger")

decorana(bio) #axis length=10.26

envn<-env[row.names(bio),]

pcnm1<-envn[,1:11] #no pcnm

envnfwda<-ordistep(cca(bio~1,pcnm1),scope = formula(cca(bio~.,pcnm1)),direction = "forward",pstep=1000)

envn1<-envn[,12:14] #Habit

envnfwda<-ordistep(cca(bio~1,envn1),scope = formula(cca(bio~.,envn1)),direction = "forward",pstep=1000)

habit<-data.frame(envn[,c(12)]) #Habit

ccah<-cca(bio,habit)

anova.cca(ccah) #0.006

ccahadj<-RsquareAdj(ccah)$adj.r.squared #0.02396

plot(ccah)

#Mantel test

habit.dis<-vegdist(habit,"euclidean")

mantel(vegdist(bio,"bray"),habit.dis)

#3.Taxonomic community for neritic and oceanic areas：

data<-read.table("allgenus.txt", header = T, row.names = 1, sep = "\t")

bio<-data.frame(t(data[,14:30]))

bio<-bio[,colSums(bio)!=0]

bio<-decostand(bio,"hellinger")

decorana(bio) #axis length=3.13

envn<-env[row.names(bio),]

pcnm1<-envn[,c(1:11)] #pcnm1+pcnm2

envnfwda<-ordistep(rda(bio~1,pcnm1),scope = formula(rda(bio~.,pcnm1)),direction = "forward",pstep=1000)

envn1<-envn[,12:14] #Habit

envnfwda<-ordistep(rda(bio~1,envn1),scope = formula(rda(bio~.,envn1)),direction = "forward",pstep=1000)

environ<-envn[, c(1,2,12)]

ccae<-rda(bio, environ)

plot(ccae)

location<-data.frame(envn[,c(1,2)])

habit<-data.frame(envn[,c(12)])

ccal<-cca(bio,location)

anova.cca(ccal)

ccah<-cca(bio,habit)

anova.cca(ccah)

winvarpart<-varpart(bio, location, habit)

winvarpart

#Mantel test

location.dis<-vegdist(location,"euclidean")

mantel(vegdist(bio,"bray"),location.dis)

habit.dis<-vegdist(habit,"euclidean")

mantel(vegdist(bio,"bray"),habit.dis)

#4.Motility community for entire SCS

data<-read.table("Biohabitcommunity.txt", header = T, row.names = 1, sep = "\t")

bio<-data.frame(t(data))

bio<-bio[,colSums(bio)!=0]

bio<-decostand(bio,"hellinger")

decorana(bio) #length=1.58

envn<-env[row.names(bio),]

pcnm1<-envn[,1:11] #pcnm1

envnfwda<-ordistep(rda(bio~1,pcnm1),scope = formula(rda(bio~.,pcnm1)),direction = "forward",pstep=1000)

envn1<-data.frame(envn[,12:15]) #Habit

envnfwda<-ordistep(rda(bio~1,envn1),scope = formula(rda(bio~.,envn1)),direction = "forward",pstep=1000)

environ<-envn[, c(1,12)]

ccae<-rda(bio, environ)

plot(ccae)

location<-data.frame(envn[,c(1)]) #pcnm1

habit<-envn[,c(12)] #Habit

ccal<-rda(bio,location)

anova.cca(ccal) #0.015

ccaladj<-RsquareAdj(ccal)$adj.r.squared

ccah<-rda(bio,habit)

anova.cca(ccah) #0.001

ccahadj<-RsquareAdj(ccah)$adj.r.squared

winvarpart<-varpart(bio, location, habit)

winvarpart

#Mantel test

location.dis<-vegdist(location,"euclidean")

mantel(vegdist(bio,"bray"),location.dis)

habit.dis<-vegdist(habit,"euclidean")

mantel(vegdist(bio,"bray"),habit.dis)

#5.Feeding habit community for entire SCS

data<-read.table("Feedcommunity.txt", header = T, row.names = 1, sep = "\t")

bio<-data.frame(t(data))

bio<-bio[,colSums(bio)!=0]

bio<-decostand(bio,"hellinger")

decorana(bio) #length=1.63

envn<-env[row.names(bio),]

pcnm1<-envn[,1:11] #PCNM1 + PCNM2 + PCNM4

envnfwda<-ordistep(rda(bio~1,pcnm1),scope = formula(rda(bio~.,pcnm1)),direction = "forward",pstep=1000)

envn1<-envn[,12:15] #Habit + Chla

envnfwda<-ordistep(rda(bio~1,envn1),scope = formula(rda(bio~.,envn1)),direction = "forward",pstep=1000)

environ<-envn[, c(1,2,4,12,15)]

ccae<-rda(bio, environ)

plot(ccae)

location<-data.frame(envn[,c(1,2,4)]) #pcnm1,2,4

habit<-data.frame(envn[,c(12,15)]) #Habit + Chla

ccal<-rda(bio,location)

anova.cca(ccal) #0.001

ccaladj<-RsquareAdj(ccal)$adj.r.squared

ccah<-rda(bio,habit)

anova.cca(ccah) #0.001

ccahadj<-RsquareAdj(ccah)$adj.r.squared

winvarpart<-varpart(bio, location, habit)

winvarpart

#Mantel test

location.dis<-vegdist(location,"euclidean")

mantel(vegdist(bio,"bray"),location.dis)

habit.dis<-vegdist(habit,"euclidean")

mantel(vegdist(bio,"bray"),habit.dis)

######correlations between communities dissimilarity and geographical distance#####

#1.intertidal vs distance#

taxonomy<-read.table("allspecieswithfeed.txt", header = T, sep = "\t")

data<-read.table("onshoresgenus.txt", header = T, row.names = 1, sep = "\t")

dis<-read.table("site.distance.on.txt", header = T, row.names = 1, sep = "\t")

dist<-dis[colnames(data),colnames(data)]

dis1<-as.dist(dist)

dis1[is.na(dis1)]<-0

disvectors<-as.vector(dis1)

data.dist<-vegdist(t(data),method = "bray")

data.distvectors1<-as.vector(data.dist)

order<-aggregate(.~Biohabit, taxonomy[,c(7:37)],sum)

data<-order[,colnames(data)]

data.dist<-vegdist(t(data),method = "bray")

data.distvectors2<-as.vector(data.dist)

order<-aggregate(.~Feeding.Type, taxonomy[,c(6,8:37)],sum)

data<-order[,colnames(data)]

data.dist<-vegdist(t(data),method = "bray")

data.distvectors3<-as.vector(data.dist)

biovsgeo<-data.frame(Taxa=data.distvectors1,Biohabit=data.distvectors2,Feedingtype=data.distvectors3,distance=disvectors)

biovsgeom<-melt(biovsgeo,id.vars=c("distance"))

ggplot(biovsgeom,aes(x=distance,y=value,color=variable))+

geom_point()+geom_smooth(method = lm)+ylim(0,1)

fit1<-lm(Taxa~distance,data=biovsgeo)

summary(fit)

cor.test(disvectors,data.distvectors1,method = 'spearman')

fit2<-lm(Biohabit~distance,data=biovsgeo)

summary(fit)

cor.test(disvectors,data.distvectors2,method = 'spearman')

fit3<-lm(Feedingtype~distance,data=biovsgeo)

summary(fit)

cor.test(disvectors,data.distvectors3,method = 'spearman')

#2.neritic vs distance#

taxonomy<-read.table("allspecieswithfeed.txt", header = T, sep = "\t")

data<-read.table("nearshoregenus.txt", header = T, row.names = 1, sep = "\t")

dis<-read.table("site.distance.near.txt", header = T, row.names = 1, sep = "\t")

dist<-dis[colnames(data),colnames(data)]

dis1<-as.dist(dist)

dis1[is.na(dis1)]<-0

disvectors<-as.vector(dis1)

data.dist<-vegdist(t(data),method = "bray")

data.distvectors1<-as.vector(data.dist)

order<-aggregate(.~Biohabit, taxonomy[,c(7:37)],sum)

data<-order[,colnames(data)]

data.dist<-vegdist(t(data),method = "bray")

data.distvectors2<-as.vector(data.dist)

order<-aggregate(.~Feeding.Type, taxonomy[,c(6,8:37)],sum)

data<-order[,colnames(data)]

data.dist<-vegdist(t(data),method = "bray")

data.distvectors3<-as.vector(data.dist)

biovsgeo<-data.frame(Taxa=data.distvectors1,Biohabit=data.distvectors2,Feedingtype=data.distvectors3,distance=disvectors)

biovsgeom<-melt(biovsgeo,id.vars=c("distance"))

ggplot(biovsgeom,aes(x=distance,y=value,color=variable))+

geom_point()+geom_smooth(method = lm)+ylim(0,1)

fit1<-lm(Taxa~distance,data=biovsgeo)

summary(fit)

cor.test(disvectors,data.distvectors1,method = 'spearman')

fit2<-lm(Biohabit~distance,data=biovsgeo)

summary(fit)

cor.test(disvectors,data.distvectors2,method = 'spearman')

fit3<-lm(Feedingtype~distance,data=biovsgeo)

summary(fit)

cor.test(disvectors,data.distvectors3,method = 'spearman')

#3.oceanic vs distance#

taxonomy<-read.table("allspecieswithfeed.txt", header = T, sep = "\t")

data<-read.table("offshoregenus.txt", header = T, row.names = 1, sep = "\t")

dis<-read.table("site.distance.off.txt", header = T, row.names = 1, sep = "\t")

dist<-dis[colnames(data),colnames(data)]

dis1<-as.dist(dist)

dis1[is.na(dis1)]<-0

disvectors<-as.vector(dis1)

data.dist<-vegdist(t(data),method = "bray")

data.distvectors1<-as.vector(data.dist)

order<-aggregate(.~Biohabit, taxonomy[,c(7:37)],sum)

data<-order[,colnames(data)]

data.dist<-vegdist(t(data),method = "bray")

data.distvectors2<-as.vector(data.dist)

order<-aggregate(.~Feeding.Type, taxonomy[,c(6,8:37)],sum)

data<-order[,colnames(data)]

data.dist<-vegdist(t(data),method = "bray")

data.distvectors3<-as.vector(data.dist)

biovsgeo<-data.frame(Taxa=data.distvectors1,Biohabit=data.distvectors2,Feedingtype=data.distvectors3,distance=disvectors)

biovsgeom<-melt(biovsgeo,id.vars=c("distance"))

ggplot(biovsgeom,aes(x=distance,y=value,color=variable))+

geom_point()+geom_smooth(method = lm)+ylim(0,1)

fit1<-lm(Taxa~distance,data=biovsgeo)

summary(fit)

cor.test(disvectors,data.distvectors1,method = 'spearman')

fit2<-lm(Biohabit~distance,data=biovsgeo)

summary(fit)

cor.test(disvectors,data.distvectors2,method = 'spearman')

fit3<-lm(Feedingtype~distance,data=biovsgeo)

summary(fit)

cor.test(disvectors,data.distvectors3,method = 'spearman')

######Dissimilarity of ciliate communities among the samples in each area######

dat<-read.table("allgenus.txt", header = T, row.names = 1, sep = "\t")

#dat<-read.table("Feedcommunity.txt", header = T, row.names = 1, sep = "\t")

#dat<-read.table("Biohabitcommunity.txt", header = T, row.names = 1, sep = "\t")

map<-read.table("allmap.txt", header = T, row.names = 1, sep = "\t")

data<-dat[,as.character(row.names(map[map$Area=="onshore",]))]

data<-data[rowSums(data)>0,]

data.dist<-vegdist(t(data),method = "bray")

data.distvectors<-as.vector(data.dist)

data<-dat[,as.character(row.names(map[map$Area=="nearshore",]))]

data<-data[rowSums(data)>0,]

data.dist<-vegdist(t(data),method = "bray")

data.distvectorw<-as.vector(data.dist)

data<-dat[,as.character(row.names(map[map$Area=="offshore",]))]

data<-data[rowSums(data)>0,]

data.dist<-vegdist(t(data),method = "bray")

data.distvectort<-as.vector(data.dist)

data.distvector<-c(data.distvectors,data.distvectorw,data.distvectort)

season<-c(rep('Onshore',78),rep('Nearshore',28),rep('Offshore',36))

bio<-data.frame(communitydissimilarity=data.distvector,season=season)

bio$season<-factor(bio$season,levels = c("Onshore","Nearshore","Offshore"))

ggplot(bio,aes(season,communitydissimilarity,fill=season))+

geom_boxplot()+

guides(fill=FALSE)+

theme(axis.text = element_text(size=15),axis.title.x=element_blank(),axis.title.y=element_text(size=15))+

ylab(label="Community dissimilarity")
